# *Mycoplasma Pneumoniae* Infection with Neurologic Complications

**Published:** 2014-07-27

**Authors:** Sevgi Yimenicioğlu, Ayten Yakut, Arzu Ekici, Kursat Bora Carman, Ener Cagrı Dinleyici

**Affiliations:** 1 Department of Pediatric Neurology; 2 Department of Pediatric Infection, Osmangazi University Medicine Faculty, Eskisehir, Turkey

**Keywords:** Mycoplasma Pneumonia, Transverse Myelitis, Gullaın-Barre Syndrome, Encephalitis

## Abstract

***Background:*** Extrapulmonary complications of *Mycoplasma pneumoniae* *(M. pneumoniae)* infection include encephalitis, optic neuritis, acute psychosis, stroke, cranial nerve palsies, aseptic meningitis and also it may be implicated in immune mediated neurological diseases such as acute demyelinating encephalomyelitis, Guillain-Barre syndrome and transverse myelitis.

***Case Presentation:*** We present five cases with acute neurological diseases after *M. pneumoniae *infection. The clinical presentations were characterized by encephalitis in 2 patients, Gullain-Barre syndrome in 2 patients, transverse myelitis in 1 patient. *M. pneumoniae *infection was detected in serum by serological method. Only two patients had respiratory symptoms preceding *M. pneumoniae* infection. Brain MRI revealed hyperintensities on corpus striatum and mesencephalon in one patient with encephalitis, the other had front parietal coalescent periventricular white matter lesions on T2 images. The patient with transverse myelitis had cervical, dorsal and lumbar scattered hyperintense lesions on T2 images. Two patients were treated with high dose steroid, the other two patients received treatment with intravenous immune globuline.

***Conclusion:*** *M. pneumoniae* may reveal different neurologic complications with different radiologic findings.

## Introduction

*Mycoplasma pneumoniae* *(M. pneumonia)* is one of the important causes of upper and/or lower respiratory tract infections during childhood. Central nervous system (CNS) related findings and complications are most commonly seen and have been described in patients with *M. pneumoniae* infections^[^^1^^,^^2^^]^. Patients suffering *M. pneumoniae* infection may have varying degrees of neurological complications at a ratio of approximately 6 to 7%^[^^1^^,^^2^^]^. Neurological manifestations include encephalitis, transverse myelitis, acute disseminated encephalomyelitis (ADEM), Guillain-Barre syndrome, and thromboembolic stroke^[^^2^^]^. The time period between the onset of respiratory symptoms and neurological symptoms varies 2 to 14 days^[^^3^^,^^4^^]^. More than 80% of patients with CNS findings have concomitant respiratory infection^[^^5^^]^.

 Herein we report five cases with various neurologic manifestations associated with *M. pneumoniae* infection.

## Case Presentation

***CASE 1: ***A 4-year-old-boy admitted with confusion, ataxia, and fatigue. He had gastroenteritis and cough for ten days. He had received acyclovir and ceftriaxone during the last four days due to suspected CNS infection. His developmental milestones were appropriate for age. There was no history of intoxication. Physical examination revealed macular rash with pale central core and darker red circle around on his trunk and lower extremities, which were not fading with pressure. He was confused. Pediatric Glasgow coma scale score was 12. He was looking senseless around and talking slowly. Muscle tone was slightly increased, deep tendon reflexes were hyperactive, Babinski was positive and clonus negative. Neck stiffness was not present. Brudzinski and Kernig signs were negative. 

 His initial laboratory evaluation was normal. Cerebrospinal fluid (CSF) examination showed no pleocytosis, protein was 16 mg/dl, and glucose 63 mg/dl (simultaneously taken blood glucose was 128 mg/dl). Ebstein-Barr virus and herpes simplex virus PCR in CSF were negative. 

**Fig. 1:**
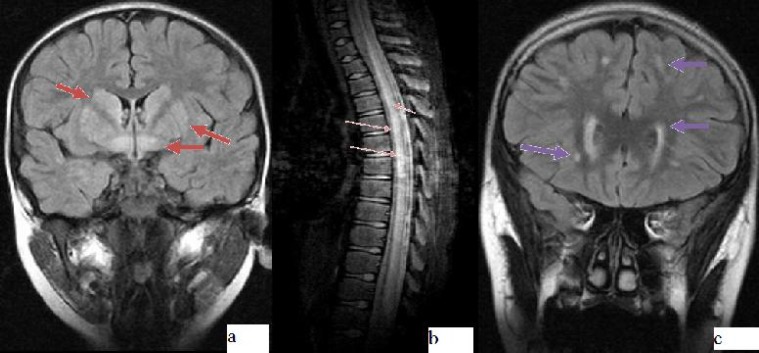
MRI findings in *M. pneumoniae* infection. **a: Case 1**:Hyperintensities at corpus striatum, mesencephalon, supratentorial sections on FLAIR images shown with red arrows. **b: Case 2**: Hyperintensities on T2 images shown with thin orange arrows**. c: Case 5**: Hyperintensities around lateral ventricules, frontoparietal region on T2 images shown with purple arrows.

 Magnetic resonance imaging revealed hyperintensities at corpus striatum, mesencephalon, and supratentorial sections on T2 weighted and FLAIR (Fluid attenuated inversion recovery) images (Fig. 1a). Electroencephalogram (EEG) was normal. The clinical signs did not subside with acyclovir, cephotaxime, vancomycin for ten days. Spasticity on his lower extremities gradually increased; he could not walk on the 14^th^ day of admission. 30 mg/kg/day methyl prednisolone for 3 days was given and then tapered slowly within two weeks. Mycoplasma antibody titers (IgM and IgG) were elevated. 15 mg/kg/day oral clarithromycine was given for ten days. After ten days he could walk with support, climb up the chair and talked faster than before. On neurological examination two years later he could walk with wide based gait, climb the chair, had positive Babinski sign, and strained Achilles tendon.

***CASE 2: ***An 11-year-old previously healthy boy developed abdominal pain ten days prior to admission. He had myalgia, could not walk and had urinary retention. He had flaccid paraparesis with areflexia, loss of pain and touch sensation up to knees. Babinski sign was positove at the right side. Cranial magnetic resonance imaging (MRI) was normal. T2 weighted images of cervical, dorsal and lumbar MRI revealed expensed spinal cord with hyperintensities compatible with myelitis and there was contrast enhancement around spinal cord (Fig. 1b). Cranial MRI was normal.

 Spinal tap showed mild increased protein (46.4 mg/dl), no pleocytosis and normal CSF glucose. Electromyography (EMG) was normal. Serologic work up confirmed *M. pneumoniae* (anti IgM and anti IgG were positive). Clarithromycine 15 mg/kg/day oral for ten days, methyprednisolone started with 30 mg/kg/day for 3 days and was tapered off within 2 weeks.

Paraparesis improved markedly, he could walk five meters without support after the first week. Had residual urine at pelvic ultrasonography supporting urinary sphincter dysfunction and micturation dysfunction one year later.

**CASE 3**

A 16-year-old boy was admitted with pain in both legs. Two days later he could not walk, he wa dependent on wheelchair. He could not grip with hands. He had quadriparesis with areflexia of legs and right arm, hyporeflexia on the left arm. Babinski sign was flexor. He could not walk. He had 33 weeks of gestational age. His developmental mile stones were appropriate for age. Cranial MRI revealed only periventricular leukomalacia supporting premature birth history. CSF examination had shown elevated protein of 151 mg/dl, no pleocytosis and, normal glucose. EMG revealed axonal demyelinating neuropathy. *M. pneumoniae* anti IgM and anti IgG was positive. He was treated with 0.4 g/kg/day of intravenous immunoglobulin (IVIG) for 5 days and, first dose 10 mg/kg/day then 5 mg/kg/day of azithromycine for 5 days. He could walk with assistance at the end of the first week of treatment and he could walk by himself at the end of first month. He was healed without neurologic deficit after 6 months follow up.

***CASE 4: ***A 15-year-old male patient admitted with calf pain and weakness. He had gastroenteritis for 7 days. He could not walk for three days. He had paraparesis with hyperreflexia of legs and Babinski sign was flexor. CSF examination showed pleocytosis (15*10 polymorphnuclear leucocytes per field), normal protein and glucose. *Campylobacter jejuni* culture was negative. EMG revealed axonal demyelinating neuropathy. *M. pneumonia* anti IgM and anti IgG was positive.

He was treated with 60 mg/kg/day vancomycin 200 mg/kg/day cefotaxime for ten days, 0.4 g/kg/day IVIG for 5 days, and 5 mg/kg/day azithromycin for 5 days. He could walk 5 meters with support at the end of the fifteenth day. He did not have any neurologic deficit after 4 months. 

**CASE 5**

A 12-year-old female patient admitted with emotional lability, forgetfulness and, headache lasting for 15 days. Headache occurred at evenings, at temples, lasted about an hour, it was throbbing. Neurologic examination was normal. CSF findings, EEG was normal. MRI revealed diffuse hyperintensities around lateral ventricles at fronto-parietal region on T2 weighted images (Fig. 1c). Magnetic resonance angiography demonstrated vasculitis of both internal carotid arteries and posterior circulation. *M. pneumoniae* anti IgM and anti IgG titers was positive. She was treated with azithromycin 5 mg/kg/day for 5 days. Her mood instability improved in 2 months. Patients’ clinical features are shown in Table1.

## Discussion

*M.pneumonia *is a respiratory pathogen recognized as a common etiology of upper tract respiratory illness and pneumonia^[^^1^^,^^2^^]^. Serologic diagnosis of Mycoplasma is difficult and problematic. The utility of IgM antibodies of *M. pneumoniae* varies with age; it is usually positive in acute infection but also may be negative in the course of acute infection or positivity may last for months^[^^1^^,^^2^^]^. Central nervous system complications have been reported with Mycoplasma infection. Cerebellar syndrome, polyradiculitis, cranial nerve palsies, aseptic meningitis, meningoencephalitis, acute disseminated encephalomyelitis, coma, optic neuritis, diplopia, mental confusion and, acute psychosis secondary to encephalitis, cranial nerve palsy, brachial plexus neuropathy, ataxia, choreoathetosis, and ascending paralysis (Guillain-Barre syndrome) are neurologic compli-cations seen with *M. pneumoniae* infection^[^^1^^]^. Encephalitis is most frequent extrapulmonary complication of *M. pneumoniae* manifested with fever, seizures, meningeal signs, ataxia, focal neurologic deﬁcits, and altered behavior, ranging from minor changes to lethargy in pediatric population^[^^2^^-^^4^^,^^6^^-^^8^^]^. Twenty percent of patients or more with CNS findings have no preceding or concomitant diagnosis of respiratory infection^[^^2^^-^^4^^,^^6^^-^^8^^]^. Among our five cases, two had respiratory symptoms at the beginning of *M. pneumoniae* infection. One of them was diagnosed as encephalitis and second one was diagnosed as Guillain-Barre syndrome with normal CSF protein levels.

 Chambert-Loir et al^[^^9^^]^ have reported a 12 years old child with acute onset of orofacial tics, motor restlessness, compulsive behavior and cerebellar symptoms.

**Table 1:** Clinical features of the 5 patients with neurologic manifestations associated with *M. pneumoniae* infection

**Case **	**Age (yr) /Gender**	**Symptoms**	**Res.** **Findings**	**MRI or CT scan**	**EMG**	**Diagnosis**	**Prognosis**
**1**	4/M	Confusion, Ataxia	+	Hyperintensities at corpus striatum and mesencephalon	-	Encephalitis	Mild spasticity at lower extremities 2 years later
**2**	11/M	Leg pain, Inability to walk, Urinate	-	Spinal MRI T2 cervical, dorsal, lumbar patchy hyperintensities	Normal	Transvers myelitis	Could walk without support 1 month later, had residual urine
**3**	16/M	Leg pain, Inability to walk	+	Periventricular leucomalacia	AND	Guillain-Barre syndrome	Foot drop 2 months later
**4**	15/M	Gastroenteritis, Leg pain, Inability to walk	-	CT normal	ADN	Guillain-Barre syndrome	Could walk without support 6 months later
**5**	12/F	Mood instability, forgetfulness, Headache, Decreased school performance	-	Fronto-parietal periventricular white matter coalescent hyperintensities	-	Encephalitis	Mood instability improved in 2 months

*M. pneumoniae:* *Mycoplasma pneumonia*; ADN: Axonal demyelinating neuropathy. CSF: Cerebrospinal Fluid; CT: Computerized tomography; EMG: Electromyography; F: Female; M: Male; MRI: Magentic resonance imaging; Res: Respiratory

Christie et al^[^^2^^]^ reported various neurologic findings with mycoplasma encephalitis. Domenech et al^[^^10^^]^ observed seizures, altered consciousness, meningeal signs, and focal motor deficits. Yis et al ^[^^12^^]^ have reported 5 patients with ocular myasthenia gravis; acute disseminated encephalomyelitis (ADEM), meningoencephalitis, transverse myelitis, and left abduscens nerve palsy. They demonstrated acute Mycoplasma infection with positive IgM and IgG titer by indirect immunofluorescence test without *Mycoplasma pneumoniae* PCR. Our second case also was diagnosed as transverse myelitis with serologic evidence of Mycoplasma antibody titers with relevant clinical findings.

 Patients with transverse myelitis may have acute motor disturbances tending to progress rapidly with leg pain, inability to walk, varying degrees of paralysis initially flaccid then evolving to spasticity when persistent; areflexia evolving to increased deep tendon reflexes, bilateral positive Babinski sign, bilateral segmental sensory changes that cannot be attributed to compression of spinal cord or another systemic disease; loss of sphincter function and MRI may reveal scattered hyperintensities of spine^[^^4^^,^^12^^,^^13^^]^.

 Several cases of *M. pneumoniae* related radiculitis and Guillain-Barre syndrome have been reported with no preceding respiratory tract infection in the literature. Flaccid paralysis or paresis with areﬂexia and various degrees of affected sensation constitutes the clinical picture. Radiculitis combined with encephalitis or myelitis may also be seen^[^^4^^]^.

 Central nervous system infections and inflammatory or autoimmune disorders may cause secondary central nervous system vasculitis. Mycoplasma may cause secondary central nervous system vasculitis in children^[14]^. Neuropsychiatric symptoms, seizures, cerebral infarction or other focal neurologic deﬁcits may be seen with CNS vasculitis secondary to infection by inﬂammation of the cerebral blood vessels by direct pathogen invasion or due to an immune-mediated response provoked by molecular mimicry, immune complex deposition, secretion of cytokines, and/or super antigen mediated responses^[14]^. One of our patients with encephalitis also had vasculitis of internal carotid artery and posterior circulation. She had neuropsychiatric manifestations.

 Neuroimaging may reveal normal ﬁndings or focal diffuse edema in cases of encephalitis or meningoencephalitis. Patchy asymmetric or diffuse signal change of gray and white matter may be seen in patients with ADEM with multifocal, asymmetric foci of high signal intensity on FLAIR and T2 weighted images. A focal infarction may be seen with *M. pneumoniae* related stroke^[^^4^^]^.

 Our two patients were diagnosed as encephalitis with intracranial imaging findings. One of the patients was admitted with confusion, ataxia and had hyperintensities at corpus striatum and mesencephalon. The second patient admitted with forgetfulness, mood disturbances, headache, and decrease in school performance was diagnosed as encephalitis with diffuse hyperintensities around lateral ventricles and bilateral frontoparietal region on MRI T2 and FLAIR weighted images with both internal carotid arteries and posterior circulation filling defects compatible with vasculitis. The other patient was diagnosed as transverse myelitis with scattered hyperintensities on cervical, thoracal and lumbar regions at MRI T2 and FLAIR studies. One patient had normal imaging findings and another one had periventricular leucomalacia related with his preterm birth.

 Treatment of neurologic complications of* M. pneumoniae* is controversial. Treatment may be adjusted according to infection mechanism such as antibiotics, corticosteroids, intravenous immunoglobulin^[^^3^^,^^12^^,^^13^^]^. Antimicrobial treatment, especially macrolides, may be sufficient for CNS involvement associated with *M. pneumoniae*, beside the beneﬁcial effect of treatment with steroids this treatment must be considered with direct invasion of CNS by the organism when other causative agents have been excluded. Plasma exchange has also been reported and seemed to be beneﬁcial. In our case series, two patients with encephalitis and transverse myelitis were treated with high dose steroid; the other two patients diagnosed as Guillain-Barre syndrome received intravenous immuneglobulin as did some authors in literature^[^^3^^,^^12^^,^^13^^]^. 

## Conclusion

Neurological manifestations associated with *M. pneumoniae *infections usually resolve completely, but they can result in chronic debilitating deﬁcits in motor or mental function with varying degrees of life threatening complications. Peripheral neurological sequelae such as radiculitis and transverse myelitis are the risk factors for chronic CNS sequels. Mental retardation, brain atrophy, hydrocephalus, epilepsy, visual changes, and global neurologic deﬁcits with brain stem dysfunction and cerebellar ataxia may be seen after encephalitis. More severe neurologic consequences were noted in 20–60% of cases in large patient series. We encountered wide based gait with mild spasticity at lower extremities after mycoplasma encephalitis, and urinary sphincter dysfunction after transverse myelitis. It is important keeping in mind *M. pneumoniae *with various neurological symptoms.
